# Prospect of Circular RNA in Hepatocellular Carcinoma: A Novel Potential Biomarker and Therapeutic Target

**DOI:** 10.3389/fonc.2018.00332

**Published:** 2018-08-23

**Authors:** Renzhi Yao, Haifan Zou, Weijia Liao

**Affiliations:** ^1^Laboratory of Hepatobiliary and Pancreatic Surgery, Affiliated Hospital of Guilin Medical University, Guilin, China; ^2^Department of Science Experiment Center, Guilin Medical University, Guilin, China

**Keywords:** circRNAs, HCC, potential values, diagnosis, therapy, prognosis

## Abstract

CircRNA, a kind of tissue specific and covalently closed circular non-coding RNA is very abundant in eukaryocyte. Generally, circRNA is generated by back-splicing of protein-coding genes' pre-mRNA. Hepatocellular carcinoma (HCC) is one of the most common malignant tumors in the world. Due to the characteristics of poor prognosis and high recurrence, the pathogenesis of HCC is highly concerned by researchers worldwide. Recent studies demonstrated that numerous circRNAs were differentially expressed in HCC tissues and normal liver tissues, which is closely related with the development and prognosis of HCC. However, the mechanism of circRNA in HCC remains unclear. In this review, we summarized the abnormal expressions of circRNAs in HCC, discussed its role, and potential mechanisms, and tried to explore the prospective values of circRNA in the diagnosis, therapy, and prognosis of HCC.

## Introduction

As a leading cause of cancer deaths globally, primary liver cancer is one of the most common malignant tumors in the world (1). There were 854,000 incident cases and 810,000 deaths worldwide in 2015 (2). The most common type of primary liver cancer, 90% of it approximately, is hepatocellular carcinoma (HCC) (3). Several risk factors, such as hepatitis B virus (HBV) and hepatitis C virus (HCV)infection, behavioral factors (alcohol, tobacco), metabolic factors as well as the aflatoxins can induce HCC (2, 4). Clinically, in the early stage of HCC, surgical resection and liver transplantation are the most effective and common treatments. However, the prognosis after treatment of HCC remains unsatisfactory. Even more frustrating are cases in which the majority of patients lost the chance of surgical therapy because they were diagnosed at the advanced stage. Therefore, elucidating the molecular mechanisms of HCC is beneficial to uncover preferable biomarkers and novel therapeutic targets of HCC.

The development of high-throughput RNA-sequencing technology has led to the discovery of numerous non-coding RNAs (ncRNAs). The number of identified ncRNA genes has already exceeded that of coding transcripts (5). Several studies have discovered that ncRNAs are involved in many pathological processes of diseases (6, 7). Circular RNA (CircRNA) is recognized as a novel ncRNA which is endogenous, abundant and steady in cells, and is related to some diseases and carcinomas (8). Although circRNA was regarded as a by-product of errant splicing, it is considered as a functional molecule nowadays (9). Recent reports revealed that circRNA could act as microRNA (miRNA) sponges to protect mRNA translation, or regulate splicing and transcription processes to affect parental genes expressions, or interact with proteins to influence their functions (10–12). More importantly, evidence has indicated that circRNA participated in HCC and played a critical role in many biological processes (13, 14). Meanwhile, circRNA might serve as a potential diagnostic biomarker and therapeutic target. However, the underlying mechanism of circRNA in HCC remains unclear till now. In this review, we concluded researches focused on the association between circRNA and HCC, and discussed the role of circRNA in HCC, including its treatment and diagnosis potency. We aimed to summarize the existing studies and provide some novel ideas and methods to excavate the mechanism of circRNA in HCC.

## General features of circRNA

Due to the rapid development and widespread application of RNA-sequencing technology, lots of ncRNAs were uncovered constantly and the number of ncRNA genes has already exceeded coding genes (15). Many kinds of ncRNAs, such as long non-coding RNA (lncRNA), miRNA, and circRNA, were identified respectively in the past two decades (16–18). CircRNA was once considered as by-products of mis-splicing of precursor mRNA (pre-mRNA) when identified in 1976 (19–23). However, over ten thousands different circRNAs have been discovered and some of their functions were also found out (24–27). At present, circRNA is widely believed to be an important kind of ncRNA and is related with human diseases, which has no caps at 5' end and no poly(A) tails at 3′ end (28). Unlike other linear RNAs, the covalently closed structure and the absence of free termini increase the resistance of circRNA to RNA exonucleases, RNase H (29) and RNase R (30) digestion. These features make circRNA more stable than linear RNAs (24, 31, 32) which may lead to the accumulation of circRNA in tissues like neurons and brain (33). But the specific functions of circRNA in these tissues are still unclear (34). Recently, circRNA has been found in almost all organisms (34). In fact, circRNA is abundant and conserved in eukaryocyte with cell type-, tissue- and stage-specificity (18, 35, 36). The abundance of circRNA exceeds the corresponding linear mRNA by over 10-fold in some cases (24). Moreover, 5–30% of circRNAs are completely conserved in human and mice (26), which provides another evidence that circRNA necessarily performs certain functions in organisms.

## Biosynthesis and types of circRNAs

That circRNAs are derived from pre-mRNAs is already known, but the exact mechanism of their biosynthesis remains unclear. Jeck et al. presented two models of exonic circRNA formation, “lariat-driven circularization,” which was also called “exon skipping” and “intron pairing-driven circularization,” as well as “ back-splicing” in 2013 (24). In 2017, Liang et al. reported that when spliceosome components were depleted or inhibited, level of circRNAs would increase while expression of linear mRNAs would decrease (37). Moreover, the inhibition of RNA polymerase II termination will extend read-through transcript to the downstream gene and lead to backsplicing, which can also increase the circRNAs level (37). Based on the previous studies, it is widely accepted that circRNA is produced by back-splicing which requires canonical splicing signal and canonical spliceosome machinery (10, 29, 38–41). After the back-splicing process, the downstream 5′ splice site of spliced RNA fragment links to the upstream 3′ splice site to form a covalently closed structure (42). Furthermore, alternative back-splice site and appropriate length of exons are also necessary (42–44). Additionally, formation of intron bracketing circRNA depends on the existence of RNA editing or hyperediting enzymes adenosine deaminases acting on RNA (ADAR) (45). During the epithelial to mesenchymal transition (EMT) process, circRNA biosynthesis can be facilitated by RNA-binding quaking (QKI) (46). Above all, biosynthesis and specificity of circRNAs can be dynamically regulated by several elements, and in different situations, the variation of circRNA components or types indicated their potential role in gene regulation indirectly.

**Figure 1:**
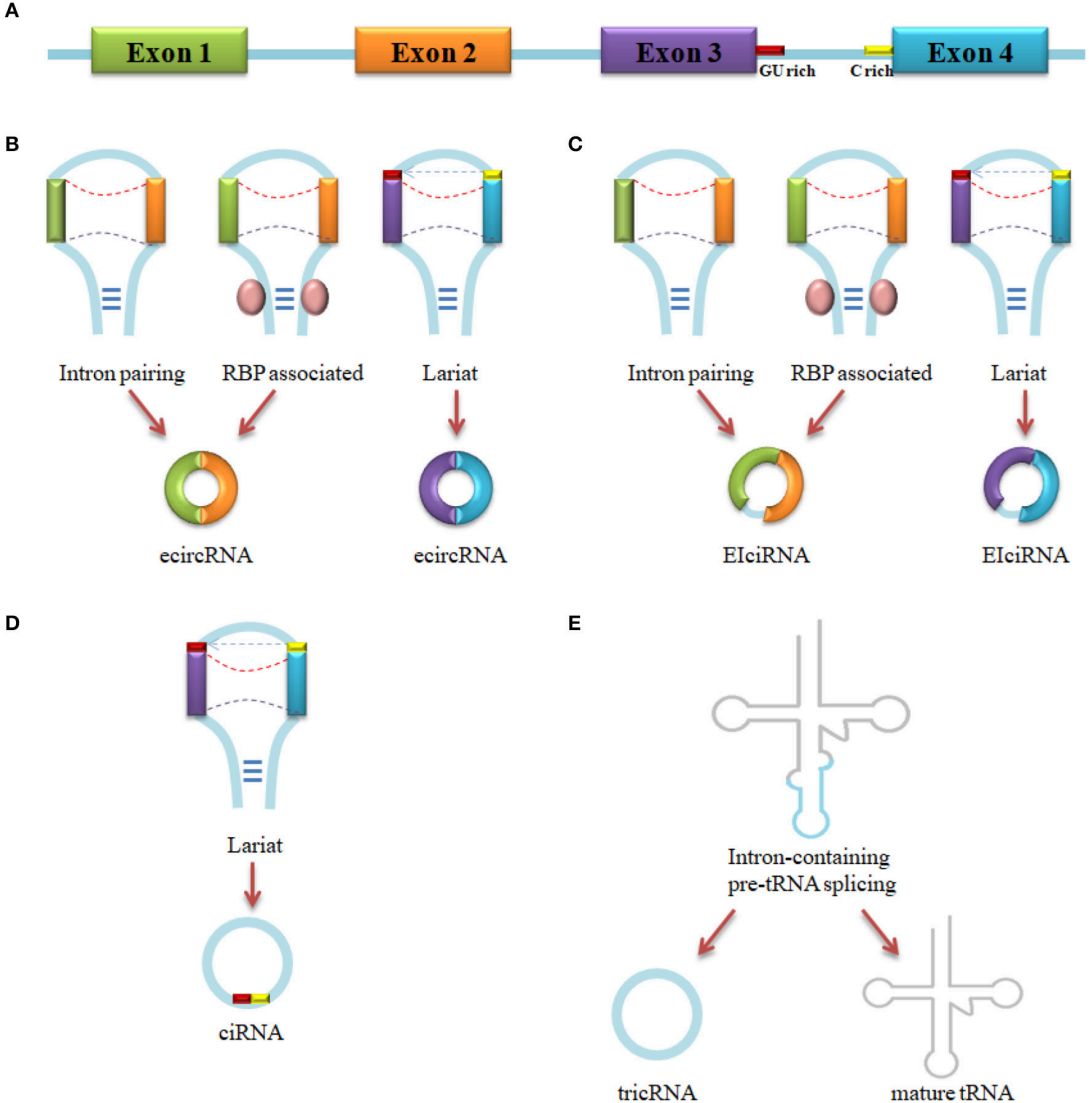
circRNA biogenesis. **(A)** Pre-mRNA transcript from parental gene. **(B)** EcircRNA biogenesis by Intron pairing-driven, RBP-associated pairing-driven and lariat-driven circularization. **(C)** EIciRNA biogenesis by Intron pairing-driven, RBP-associated pairing-driven and lariat-driven circularization. **(D)** CiRNA biogenesis by lariat-driven circularization. **(E)** TricRNA biogenesis by intron spliced from pre-tRNA.

Up to now, four types of circRNAs have been identified: exonic circRNA (ecircRNA) (24), intronic circRNA (ciRNA) (36), exon-intron circRNA (EIciRNA) (11), and tRNA intronic circRNA (tricRNA) (47). The vast majority of circRNAs is ecircRNA (48). As is shown in Figure 1, ecircRNA and EIciRNA are produced by intron pairing-driven, RBP-associate pairing-driven and lariat-driven circularization, while ciRNA is generated by lariat-driven circularization and tricRNA is formed by pre-tRNA intronic splicing. Intron pairing-driven circularization is accomplished by the direct base-pairing of the introns flanking complementary sequences or inverted repeats (38, 49). RBP-associate pairing-driven circularization is led by the pairing of two flanking introns which are closed to the flanking intronic reverse complementary sequences (e.g., Alu element) (24). The RBP-associate pairing-driven circularization process requires the involvement of RNA binding protein (RBP), like *muscleblind*(MBL) (10), QKI (46), heterogeneous nuclear ribonucleoprotein (hnRNP) and serine/arginine proteins (50), but the process can be inhibited by the existing of ADAR1 and DHX9 (45, 51). Lariat-driven circularization forms a lariat which contains exons through exon skipping event, or a lariat that contains a 7 nt GU-rich element near the 5′ splice site and a 11 nt C-rich element closed to the branchpoint site consensus motif (36, 43, 52). In the present, one study has demonstrated a novel model that an intron-containing pre-tRNA could be cleavaged by tRNA splicing endonuclease (TSEN) complex at the bulge-helix-bulge (BHB) sequence motif, then the exon go halves and the intron termini was ligated to form a tricRNA (47). About 30–40 base-pairings could promote the formation of circRNA, but low-complexity sequences or subtle distortions in the hairpin between the repeats would inhibit the generation (53).

Not all circRNAs in eukaryote are generated based on base-pairing. For instance, flanking intronic complementarity is not crucial for *Drosophila* circularization, instead, extended length of flanking upstream, and downstream introns are the determinant (54). Furthermore, only a very small part of circRNAs in rice forms from base-paring-driven back-splicing (55). Interestingly, an expression vector inserted with a 20 nt flanking intron which is lacking of inverted repeats can also produce circRNA (29).

Another study found that general splicing factors can enhance or inhibit back-splicing through the combination with *cis*-acting splicing regulatory elements (SREs) (56). Such *cis*-elements can be recognized by RBP (10, 46), an assistance of portions of circRNAs generation, to act as enhancers or silencers of exonic splicing (39). Above all, circRNAs formation might be regulated by many factors like *cis*-elements or trans-acting elements in gene locus, especially in intron, which means that circRNAs generation is a complicated process and what we have found so far is just a tip of the iceberg.

## Functions of circRNAs

Studies on circRNAs demonstrated that different types of circRNAs have different features. EIciRNAs and ciRNAs are located in the nucleus mostly and they can enhance expression of parental genes through binding to polymerase II (11, 36). The majority of ecircRNAs are located in the cytoplasm. Portions of them combine with miRNA and function as a miRNA sponge to regulate the function of miRNA (28, 57–60), and the other can intervene in the RNA process to regulate the replication of virus and viroid (61).

### miRNA sponge

Several studies have revealed that circRNAs could be targeted by RNA interference, which suggested that circRNAs might function as miRNA sponges or competitive endogenous RNAs (ceRNAs) to regulate the expression of miRNA target genes (24). MiRNA, a class of ncRNA of approximately 22 nt in length, can directly bind to the matched 3′-untranslated regions (3′-UTR) of target genes via the seed sequence and regulate the target genes at post-transcription (62). The following studies demonstrated that some circRNAs contain miRNA target sites so that circRNAs can regulate the translation of mRNA indirectly (9). The most typical example is that Cdr1as, an abundant circRNA in the mammalian brain, which contains more than 70 conserved miR-7 binding sites and a perfectly complementary miR-671 site to the entire mature miR-671 sequence (63). Losses of Cdr1as can downregulate levels of miR-7 and miR-671 in brain specifically, and losses of Cdr1as-miRNAs interaction can lead to an altered neuronal activity which might cause the sensorimotor deficits and the neuropsychiatric phenotype. Furthermore, de-repression of immediate early genes (IEGs) was also observed in that case. Since most of IEGs are proto-oncogene, such as *c-fos* (64), de-repression of these genes may result for the development of cancer. Similar cases were also observed in breast cancer. CircTCF25 combines with miR-103a-3p and miR-107 can lead to the upregulation of 13 genes related to proliferation, metastasis and invasion. The overexpression of circTCF25 suppresses miR-103a-3p and miR-107 expression and enhances the expression of CDK6 to promote cell proliferation and metastasis (65). Another circRNA, circITCH, which is overexpressed in breast cancer, can function as miR-17 and miR-224 sponges and regulate p21 and PTEN genes to inhibit the development of breast cancer (66). However, circRNAs with miRNA binding sites are rarely seen in human and mice, suggesting that the majority of circRNAs cannot function as miRNA sponges (11, 26, 30, 55, 67). Despite this, the existing results remarkably demonstrate that the circRNA-miRNA interaction is crucial for organisms.

### Transcription regulation

Although most of the circRNAs are generated from exons and distributed in cytoplasm, ciRNA, and EIciRNA, absent of miRNA binding site, were being found located in the nucleus abundantly and function as transcriptional regulatory *cis*-element (35, 68). Inhibition of ciRNA expression leads to the downregulation of parental genes. For example, knockout of *ci-ankrd52* and *ci-sirt7*, which regulate the transcription of their parental genes in *cis* or *trans* via interact with the elongating RNA polymerase II (Pol II) complex, can lead to the downregulation of transcription of their parental genes ANKRD52 and SIRT7 (35, 36). Moreover, knockout of EIciEIF3J and EIciPAIP2, two kinds of EIciRNAs, can reduce the transcription of their parental genes (11). The mechanism of the EIciRNA inhibition probably is that EIciRNA can interact with the Pol II and the promoter of their encoding gene, U1 small nuclear ribonucleoprotein (U1 snRNP) through U1 snRNP binding sites, to enhance their parental genes transcription.

Therefore, though ciRNA and EIciRNA cannot function as miRNA sponges, they can regulate gene expression and transcription via other pathways like playing *cis*- or *trans*-acting elements roles, which is indicated in the present findings.

### Protein regulation

As is described above, many circRNAs are predicted to interact with RBPs (10, 11, 28, 34). Thus circRNA may have the potential to regulate protein functions via bind, store, sort, and sequester proteins to particular subcellular locations, and act as dynamic scaffolding molecules that modulate protein-protein interactions (12). Latest studies showed that circ-Amotl1 could interact with c-myc, STAT3, PDK1, and AKT1 proteins to affect the expressions of their target genes through facilitating the nuclear translocation of such proteins (69–71). Overexpression of circ-Foxo3 can prevent cell cycle progression by forming circ-Foxo3-p21-CDK2 ternary complexes via binding to CDK2 and p21 proteins (72), and can also bind and sequester ID-1, E2F1, FAK, and HIF-1α proteins to inhibit their ability of anti-aging and anti-stress (73). In addition, a recent study showed that circRNAs can affect translation process by competitive binding to RBP with their parent mRNA (74). All the evidences demonstrated that circRNA were more likely to regulate the function of proteins by acting as a scaffold of protein complexes instead of binding to a single protein simply.

### Translation into protein

Due to the containment of open reading frame (ORF), the protein translation potential of circRNA has already been discovered. As early as 1998, Perriman et al. had reported that circular mRNA which contained a complete ORF of GFP could directly produce GFP in *Escherichia coli* (75). More studies have found that ecircRNAs containing of internal ribosome entry sites or prokaryotic binding sites have protein-coding ability both *in vivo* and *in vitro* (39, 76). Recent study have shown exactly that circ-ZNF609, an endogenous circRNA contains an ORF, as the linear transcript, from the start codon to in-frame STOP codon, can be translated into a protein in a splicing-dependent and cap-independent manner in myogenesis (77). Even though many circRNAs have been predicted with the capacity of protein translation (78), there are no enough evidences to prove that circRNAs can function as mRNA (41).

## Circrnas in hepatocellular carcinoma (HCC)

As a malignant tumor with high mortality rate, liver cancer is one of the most common malignant tumors in the world with poor prognosis (79). Statistics show that over 854,000 people are diagnosed with liver cancer, and meanwhile, more than 810,000 died of this disease, ranking second of all cancer deaths, fifth of cancer morbidity and third of cancer mortality (2, 80–83). Up to 90% of primary liver cancers are HCC (84). It is well known that HCC has poor prognosis, high degree of malignancy and high recurrence rate, and is resistant to many chemotherapeutic agents (85, 86). Sadly, the tumor-related cells of HCC usually metastasized via blood or lymphatic, and the malignant metastasis can hardly be controlled as long as it occurs (87). Furthermore, the lack of effective clinical prediction, diagnosis and treatment methods of HCC urges scientists to discover better tumor markers. Recently, researchers have found that the imbalance expression of ncRNAs, especially circRNAs, is more common in HCC and is closely related with the development of the disease. Such phenomenon indicates that scientists may be able to illuminate the mechanism of HCC and find out the prognosis and therapeutic targets for HCC in the circRNAs field. All circRNAs in HCC and mentioned in this review were listed in Table 1.

**Table 1:** CircRNAs in HCC.

**Name given in paper**	**CircBase ID**	**Gene symbol**	**Type of study**	**Functions**	**Potential role**	**References**
has_circ_0005075	has_circ_0005075	EIF4G3	Human	miRNA sponge and promoter	Diagnosis	(14)
Cdr1as/ciRS-7	hsa_circ_0001946	CDR1	Human and cell line	miRNA sponge and promoter	Diagnosis and therapeutic target	(88)
			Human	miRNA sponge	Therapeutic target	(89)
circRNA_100338	Not provided/not traceable	SNX27	Human and cell line	miRNA sponge and promoter	Diagnosis and therapeutic target	(90)
hsa_circ_0067934	hsa_circ_0067934	PRKCI	Human and cell line	miRNA sponge	Therapeutic target	(91)
hsa_circ_0005986	hsa_circ_0005986	PRDM2	Human and cell line	miRNA sponge	Prognostic predictor and therapeutic target	(92)
circMTO1	Not provided/not traceable	MTO1	Human, cell line, and nude mice	miRNA sponge and suppressor	Prognostic predictor and therapeutic target	(93)
hsa_circ_0004018	hsa_circ_0004018	SMYD4	Human and cell line	miRNA sponge	Diagnosis	(94)
cSMARCA5	hsa_circ_0001445	SMARCA5	Human, cell line, and nude mice	miRNA sponge and suppressor	Prognostic predictor and therapeutic target	(95)
hsa_circ_0046367	hsa_circ_0046367	FASN	Human and cell line	miRNA sponge	Therapeutic target	(96)
circC3P1	Not provided/not traceable	C3P1	Human, cell line, and nude mice	miRNA sponge and suppressor	Prognostic predictor	(97)
hsa_circ_0001649	hsa_circ_0001649	SHPRH	Human and cell line	miRNA sponge	Prognostic predictor	(13)
circ-ITCH	Not provided/not traceable	ITCH	Human	suppressor	Prognostic predictor	(98)
circZKSCAN1	hsa_circ_0001727	ZKSCAN1	Human, cell line, and nude mice	Suppressor	Diagnosis	(99)

### Function as miRNA sponges

The observation in the structure of circRNAs has shown that circRNAs have numerous miRNA binding sites which assist circRNA in interacting with miRNA (61). Further studies proved that circRNAs could regulate the expression of parental gene via interact with miRNA (100, 101). For example, the upregulation of has_circ_0005075 expression correlated with HCC tumor size, which indicated that has_circ_0005075 might promote the growth of tumor, and showed good diagnostic potential (AUROC = 0.94). Moreover, has_circ_0005075 was predicted to get involved in cell adhesion by using GO and pathway analysis, which was strongly associated with cell proliferation, invasion, and metastasis in HCC. The effect of biological functions speculated that has_circ_0005075 could regulate some components of the tumor cell membrane. Furthermore, has_circ_0005075 was found to interact with four miRNAs potentially, including hsa-miR-23b-5p, hsa-miR-93-3p, hsa-miR-581, and hsa-miR-23a-5p, in order to inhibit the expression and functions of those miRNAs. According to the results above, we have the reason to believe that high expression level of has_circ_0005075 in HCC is correlated with tumor progression and has_circ_0005075 might be utilized as a novel biomarker for HCC (14).

Another study reported a well-known circRNA, Cdr1as, also called ciRS7, which contained more than 70 conserved miR-7 binding sites and a perfectly complementary miR-671 site, was significant upregulated in HCC (88). Knockdown of Cdr1as could suppress the proliferation and invasion of HCC cell. Meanwhile, the expression level of miR-7 was also suppressed. Overexpression of miR-7 could inhibit the expression of its direct target genes *CCNE1* and *PIK3CD*, which were inhibited by Cdr1as silencing either. These results suggested that Cdr1as might act as an oncogene partly through targeting miR-7 in HCC (88). Interestingly, Xu et al. presented an inverse result that Cdr1as was downregulated in HCC and the expression of Cdr1as was significantly correlated with age < 40 years, serum AFP ≥400 ng/μl, hepatic MVI and two miR-7-targeted genes, *PIK3CD* and *p70S6K* (89). A similar conclusion was that the expression of Cdr1as was related to miR-7 target gene *PIK3CD* and Cdr1as could function as miR-7 sponge. Taken together, one thing can be confirmed is that the expression level of Cdr1as is closely related to HCC. Whether Cdr1as can serve as a promising HCC biomarker requires further validation due to the unclear function of Cdr1as in HCC.

The upregulation of circ_100338, selected from circRNA microarray analysis, was closely related to low cumulative survival and positively correlated with metastasis progression of HCC patient with HBV infection (90). As a sponge of miR-141-3p, circ_100338 can be counteracted by miR-141-3p and inhibited cell metastasis progression of HCC. The inhibition of invasive capacity caused by miR-141-3p overexpression can be restored by co-expressing of miR-141-3p and circ_100338 in MHCC97H cells. Similarly, the enhancement of migratory and invasive ability of MHCC97H cell induced by circ_100338 can be rescued by miR-141-3p upregulation. Such results demonstrated that circ_100338 has the potential as a novel biomarker for diagnosis and patient survival estimation in HBV-related HCC patients (90).

Several signaling pathways have been reported to be involved in pathological mechanism of HCC (102). FZD5 has been reported as a co-receptor and a positive activator of Wnt/β-catenin signaling pathway (103, 104). A recent study showed that circ_0067934, whose expression was upregulated in HCC, could upregulate the Wnt/β-catenin signaling pathway through functioning as a miR-1324 sponge. MiR-1324 could target the 3′-UTR of FZD5 to impair the activation of Wnt/β-catenin signaling pathway. Knockdown of circ_0067934 or miR-1324 overexpression can both downregulate FZD5 expression and suppress the activation of Wnt/β-catenin signaling pathway while significantly suppress cell proliferation, migration and invasion of Hep3B and HuH7 cells. The results demonstrated that the circ_0067934/miR-1324/FZD5/Wnt/β-catenin signaling axis might serve as a promising target for HCC intervention. Moreover, cell apoptosis was also observed in this case (91).

Further study revealed that hsa_circ_0005986, a target of miR-129-5p, was significantly lower than normal tissues compared to HCC tissues (92). Statistic analysis showed that low expression level of hsa_circ_0005986 was correlated with chronic hepatitis B family history, tumor diameters, microvascular invasion and Barcelona Clinic Liver Cancer (BCLC) stage, while experiments showed that the downregulation of hsa_circ_0005986 liberated miR-129-5p and decreased the expression level of *Notch1* mRNA, which was validated as a target of miR-129-5p previously (105). Interestingly, the downregulation of hsa_circ_0005986 improved cell proliferation by promoting the G0/G1 to S phase transition. The previous experiment has implied that the phase transition was regulated by G0/G1 switch gene 2 (G0S2), which might be a significant regulator of HCC cells proliferation via activating PPARα, a key regulator of fatty acid catabolism in the liver (106), therefore hsa_circ_0005986 may be involved in HCC progression through regulating cell proliferation of HCC.

A remarkable study analyzed the expression profile of human circRNAs in HCC tissues and found a circRNA named circMTO1(*hsa_circRNA_0007874/hsa_circRNA_104135*), which was significantly downregulated in HCC tissues, could act as a sponge of oncogenic miR-9 and was correlated with poor prognosis of HCC patients (93). MiR-9 has been reported upregulated in HCC frequently and is closely related to poor prognosis of HCC patients (107, 108). Mechanistically, circMTO1 bound with miR-9 in the cytoplasm could upregulate p21 expression and suppress HCC progression. p21 has been identified as a tumor suppressor and a target of miR-9 (109–112). Knockdown of circMTO1 enhanced cell proliferation and invasion, as well as reduced cell apoptosis and both p21 mRNA and protein expressions, while circMTO1 overexpression promoted apoptosis and upregulated the mRNA and protein expression of p21. Moreover, miR-9 overexpression showed a similar effect on tumor cells as circMTO1 silencing. The test of miR-9 inhibition in cricMTO1-silencing cells revealed that miR-9 inhibitor could restrict the promotion effect induced by circMTO1 knockdown in HCC cells, which demonstrated that circMTO1 could sponge miR-9 and suppress miR-9 oncogenic effect via circMTO1/miR-9/p21 axis. In addition, the knockdown of circMTO1 inhibited p21 and the downstream CDK2 expressions, while MMP2 and PCNA, markers of invasion and proliferation respectively, were upregulated *in vivo*, which indicated that circMTO1 could inhibit HCC progression both *in vivo* and *in vitro* (93). Consequently, circMTO1 may serve as a prognosis predictor and therapeutic target for HCC potentially.

Another microarray analysis revealed that a downregulated circRNA transcribed from *SMYD4* and named hsa_circ_0004018, was correlated with serum AFP level, tumor diameters, differentiation, BCLC stage and TNM stage (94). An interesting result showed that hsa_circ_0004018 possessed a HCC-stage-specific expression feature. *SMYD4* has been reported as a potential tumor suppressor and was involved in carcinogenesis (113–115), which prompted that, as a transcript of *SMYD4*, hsa_circ_0004018 might be involved in the development of HCC. Moreover, the sensitivity of hsa_circ_0004018 was superior to that of AFP, which means hsa_circ_0004018 may play a crucial role in HCC monitoring. Further bioinformatics prediction indicated that hsa_circ_0004018 harbored five miRNAs seed sequences and might play an important role in HCC carcinogenesis and metastasis via interacting with miR-30e-5p/miR-626-MYC (94).

Due to the regulation of DExH-Box Helicase 9 (DHX9), cSMARCA5 (hsa_circ_0001445) was downregulated in HCC tissues while *SMARCA5* mRNA and protein were upregulated (95). DHX9 is an abundant nuclear RNA helicase which can bind to inverted-repeat Alu elements and inhibit the formation of circRNAs (51). Silencing of DHX9 at the helicase active site could upregulate the expression of cSMARCA5, which indicated that the regulation of circRNA depended on the helicase activity of DHX9. Further experiments showed that cSMARCA5 could inhibit the proliferation and migration of HCC cells through promoting a tumor suppressor TIMP3 by sponging miR-17-3p and miR-181b-5p. Moreover, low expression of cSMARCA5 was also considered as an independent risk factor of overall survival (OS) and recurrence-free survival (RFS) of HCC patients after hepatectomy. The results demonstrated that cSMARCA5 might function as a tumor suppressor via DHX9-cSMARCA5-miR-17-3p/miR-181b-5p-TIMP3 axis pathway to inhibit the growth and metastasis of HCC; while its parental gene *SMARCA5* may be a tumor promoter (95), which hinted that *SMARCA5* and cSMARCA5 levels could act together as a biomarker for HCC.

As is well-known, hepatic steatosis is involved in the HCC disease progression (116). circRNA_0046367, a circRNA which was filtered from ncRNA databases and acted as an endogenous modulator of miR-34a, was dramatically decreased in high-fat-induced steatosis HCC cells (96). The downregulation of circRNA_0046367 signified the loss of inhibition of miR-34a/PPARα interaction and led to lipid peroxidative damage, thereby reducing hepatic steatosis. PPARα is a ligand-activated transcription factor and underlies the fatty acid metabolism (117). PPARα downregulation decreased PPARα-mediated lipid metabolism and led to hepatocellular steatosis, which indicated that circRNA_0046367/miR-34a/PPARα axis might be involved in lipoxidative and reduce hepatic steatosis-inducing HCC (96). Moreover, other same results have been observed in circRNA_0046366 (118).

Through analyzing a circRNA-sequencing dataset (GSE77661), circC3P1 was filtered as one of the most downregulated circRNAs in HCC (97). The circC3P1 expression was negatively correlated with TNM stage, tumor size and vascular invasion through analyzing clinical data, and the analysis also showed that the higher expression of circC3P1, the lower survival rate of HCC patients. Further experiments investigated that circC3P1 overexpression markedly suppressed the proliferation, migration and invasion of HCC cells *in vivo* and *in vitro*, which indicated that circC3P1 could serve as a tumor suppressor in HCC. In addition, circC3P1 could promote PCK1, a key enzyme of gluconeogenesis, which was downregulated in HCC, and its decrease by sponging miR-4641 in HCC might lead to hepatocarcinogenesis (119). The inhibition of HCC cells characteristics caused by circC3P1 overexpression would be abolished by PCK1 silencing. Such results revealed that circC3P1/miR-4641/PCK1 axis could regulate the growth and metastasis of HCC and serve as a prognostic biomarker for HCC patients (97).

Has_circ_0001649 was produced from *SHPRH* and was found significantly downregulated in HCC tissues (13). The level of has_circ_0001649 was related to tumor size and tumor embolus. In addition, silencing of has_circ_0001649 resulted in decrease of MMP9, MMP10, and MMP13, which could increase the metastasis of HCC (120–123), indicating that downregulation of has_circ_0001649 was positively correlated with metastasis and growth of HCC. Further ROC analysis showed that has_circ_0001649 was associated with poor prognosis of HCC and might serve as a biomarker for HCC. Sequence analysis revealed that has_circ_0001649 had the potential for protein sponge or transcription regulator with containing of one U2 auxiliary factor (U2AF) binding site, five eukaryotic initiation factor 4A-III (EIF4A3) binding sites and one regulator of nonsense transcripts 1 (UPF1) binding site to participate in HCC development and progression. Moreover, has_circ_0001649 was also regarded as a miRNA sponge through bioinformatics analysis. The analysis showed that has_circ_0001649 possessed potential binding sites for at least eight miRNA (13). This report demonstrated that the functions of circRNAs were complicated. It may function as protein sponge and miRNA sponge at the same time to regulate the same or different features of cancers.

### Function as an inhibitor or promoter of HCC

It has been reported that circ-ITCH suppressed several cancers proliferation through inhibiting the Wnt/β-catenin signaling pathway (57, 124, 125). After analyzing numerous cases and conducting a genetic association study together with an epidemiological follow-up study, Guo et al. found that circ-ITCH expression in HCC tissues was lower than that of the adjacent tissues and the high expression of circ-ITCH was associated with favorable survival of HCC (98). Further analysis showed that two single nucleotide polymorphisms (SNPs) sites-rs10485505 and rs4911154 in circ-ITCH were closely related to the increased risk of HCC. These results demonstrated that circ-ITCH might function as an inhibitor of HCC (98). More than that, genetic variation of circ-ITCH might act as a susceptible biomarker for prediction and screening of HCC.

*ZKSCAN1* is a kind of zinc family gene, which is upregulated and related to the proliferation of tumor cells in several cancers (126). Exon 2 and 3 of *ZKSCAN1* spliced together to form a circRNA termed *circZKSCAN1* (hsa_circ_0001727), which is abundant in human brain and liver (53). In the present study, both *ZKSCAN1* mRNA and *circZKSCAN1* were observed obviously downregulated in HCC (99). The downregulation of *ZKSCAN1* mRNA was correlated with tumor size while *circZKSCAN1* levels affected tumor number, cirrhosis, vascular invasion or microscopic vascular invasion and tumor grade. Further experiments indicated that silencing or overexpression of both RNA enhanced or suppressed cell proliferation, migration and invasion respectively *in vivo* and *in vitro*. Meanwhile, no mutual interference was observed during the experiments above, which meant that these two homologous RNAs might regulate different features in HCC. RNA-seq supported the hypothesis that *ZKSCAN1* mRNA regulated cellular metabolism and *circZKSCAN1* was involved in cancer-associated signaling pathway (99). Consequently, *ZKSCAN1* mRNA and *circZKSCAN1* might function as a HCC inhibitor via interacting with each other to suppress metabolism, apoptosis, proliferation, and metastasis of HCC.

Interestingly, many circRNAs served as miRNA sponge can also be considered as tumor suppressors of HCC, such as cSMARCA5 (95), circMTO1 (93), and circC3P1 (97), while some kinds of circRNAs can act as tumor promoters, like has_circ_0005075 (14), circ_100338 (90), etc. Such phenomenon manifested that the mechanism of circRNAs in HCC was complicated. A circRNA may act as a tumor suppressor or promoter with or without sponging miRNA, binding to proteins or participating in transcription or post-transcription. This suggests that one circRNA may affect different HCC progressions through different pathways, which means circRNAs may have the potential to be a biomarker or therapeutic target of HCC.

## Conclusion and prospect

With the continuous advancement of researches, the functions of circRNA were constantly being revealed, from the by-products of erroneous splicing to the small molecule with important biological functions. Like other ncRNAs, numerous circRNAs were validated to participating in HCC progressions. In this review, we demonstrated that circRNA, an abundant, stable, conserved kind of ncRNA with diversity structures, has great potential for regulation, prediction, diagnosis, and a therapeutic target in diseases, especially in HCC. However, we cannot explain how circRNA participates in HCC development clearly till now due to its complicated mechanism. Importantly, how and what circRNA do in cells are also waiting for discovery (34). Moreover, the particular characteristics of circRNAs structure and insufficient techniques limit the probe into the functions of circRNA (127). Hence, the development and use of appropriate techniques to elucidate the molecular mechanisms of circRNAs in HCC and its regulatory networks are the top priority for future studies. For example, the novel gene editing technology, CRISPR system, may help us understand what circRNAs can do *in vitro*. CasRX was identified as a RNA-guided, RNA-targeting CRISPR system, which is more efficient and more specific than RNA interference (128). CasRX is a member of Cas13d family and functions as a programmable RNA-binding module for efficient target of cellular RNA (128), which indicates that it has the potential for modulating circRNA and regulating disease development. In addition, nuclease-deactivated Cas9 (dCas9) was used to labeling endogenous genomic loci (129). Hence, using modified CasRX to label a certain circRNA and figuring out its location and dynamic change in cells may help researchers comprehend the mechanism of circRNA. Moreover, with the capacity of binding specific proteins, circRNA may act as selective markers for flow cytometry.

At present, most of the identified circRNAs in HCC functions as miRNA sponges and/or cancer suppressors or promoters. However, numerous studies have reported that circRNA could regulate cancer development via binding to crucial proteins directly (72, 73). What's more, it has the potential for translating into proteins or peptides to participate in cancer progression (77, 78). In addition, ciRNA, and EIciRNA could interact with polymerase II and U1snRNP to regulate the transcription of their parental genes (11). Therefore, whether circRNA participates in the process of HCC at transcription level or post-transcription level, or whether it functions through translating into proteins directly is still kept in mystery. If so, how circRNA gets involved in those processes? If not, are there any other pathways for circRNA to affect HCC?

A novel study revealed that, in addition to cells, abundant circRNAs were also found in exosomes, blood, and saliva (130–132). Furthermore, serum exosomes-circRNA might be able to distinguish patients with cancer from healthy individuals, indicating that circRNA might serve as a circulating biomarker for cancer diagnosis. Moreover, because of exosomes are secreted by all cells and circulate in the blood (133, 134), circRNA might influence distal cells in the way that it is transported by exosomes. A study found that a hepatoma cell-secreted exosomal miRNA, miR-103, could affect HCC metastasis, and enhance vascular integrity (135). In addition, long non-coding RNA (lncRNA) *ANRASSF1* was identified to downregulate the expression of tumor suppressor gene *RASSF1A* through combining with DNA to produce lncRNA/DNA hybrid and recruiting SUZ12 protein at promoter region of *RASSF1A* to suppress the expression of *RASSF1A* (136). However, as a member of ncRNA, the stable circRNA affects HCC progression in the way of secreting into blood circulation to play a crucial role in cancer detection potentially, or interacting with cancer-related genes directly to regulate critical genes of HCC is still unknown.

Consequently, circRNAs could be involved in the development of disease, tissue development and gene regulation, and some of them even play a crucial role in carcinogenesis (137). However, with a small number of consolidated researches, we can merely draw the conclusion that circRNA participates in the development of HCC, but not the conclusion that circRNA must plays a key role in the process. It is certain that with the continuous development of techniques and continuous advancement of studies, like RNA-seq for ncRNAs (138, 139) and other experiment techniques, the role of circRNA in the development of HCC will be gradually revealed. Finally, we will illuminate the network of circRNA in HCC or in other diseases and determine whether circRNA is the key factor in the disease progression.

## Author contributions

RY wrote the manuscript. HZ collected and analyzed the published studies. WL conceived and polished the article.

### Conflict of interest statement

The authors declare that the research was conducted in the absence of any commercial or financial relationships that could be construed as a potential conflict of interest.

## Abbreviations

EMT: epithelial to mesenchymal transition
ADAR: adenosine deaminases acting on RNA
QKI: RNA-binding quaking
ES: exon skipping
A5SS: alternative 5′ splicing site
A3SS: alternative 3′ splicing site
RBP: RNA binding protein
MBL: *muscleblind*
hnRNP: heterogeneous nuclear ribonucleoprotein
DHX9: DExH-Box Helicase 9
TSEN: tRNA splicing endonuclease
BHB: bulge-helix-bulge
SREs: splicing regulatory elements
ceRNA: competitive endogenous RNA
3′-UTR: 3′-untranslated region
IEGs: immediate early genes
CDK2 and CDK6: cyclin-dependent kinases 2 and 6
PTEN: phosphate and tension homology deleted on chromosome ten
STAT3: signal transducers and activators of transcription 3
PDK1: 3-phosphoinositide-dependent protein kinase-1
AKT1: AKT serine/threonine kinase 1
ID-1: inhibitor of DNA-binding protein 1
E2F1: E2F transcription factor 1
FAK: focal adhesion kinase
HIF-1α: hypoxia inducible factor-1
AUROC: area under the receiver operating characteristic curve
AFP: alpha fetoprotein
MVI: microvascular invasion
FZD5: frizzled class receptor 5
TIMP3: metalloproteinase inhibitor 3
PPARα: peroxisome proliferator-activated receptor α
PCK1: Phosphoenolpyruvate carboxykinase 1
CRISPR: clustered regularly interspaced short palindromic repeats.
